# Joint aortic root segmentation and landmark localization on intraoperative fluoroscopy for TAVI guidance

**DOI:** 10.3389/fcvm.2026.1886469

**Published:** 2026-07-15

**Authors:** Nikita V. Laptev, Olga M. Gerget, Julia K. Panteleeva, Mikhail A. Chernyavsky, Viacheslav V. Danilov

**Affiliations:** 1 Siberian State Medical University, Tomsk, Russia; 2 Institute of Control Sciences of Russian Academy of Sciences, Moscow, Russia; 3 Almazov National Medical Research Center, Saint Petersburg, Russia; 4 Pompeu Fabra University, Barcelona, Spain

**Keywords:** aortic root segmentation, fluoroscopy, landmark localization, multitask learning, TAVI

## Abstract

Accurate intraoperative delineation of the aortic root and localization of key anatomical landmarks during transcatheter aortic valve implantation (TAVI) remain difficult because fluoroscopy provides low soft-tissue contrast and is frequently degraded by motion and overlap from catheters and delivery systems. This study developed and validated a multitask deep learning model for simultaneous aortic root segmentation and landmark localization on fluoroscopic images to support image-guided TAVI. A retrospective dataset of 2,895 fully anonymized fluoroscopic frames from 83 patients who underwent TAVI between 2018 and 2024 was used. Expert annotations included binary masks of the contrast-enhanced aortic root and four anatomical landmarks: two aortic annulus points (AA1, AA2) and two sinotubular junction points near the coronary ostia (STJ1, STJ2). We developed BoundaryAwareMANet (BAMNet), a multitask architecture combining an EfficientNet-V2 encoder, an MA-Net-inspired decoder, a coordinate-aware landmark head, and an auxiliary boundary-guidance pathway. Model performance was evaluated using patient-level five-fold cross-validation. Across five folds, BAMNet achieved Dice 0.916±0.011, IoU 0.850±0.018, and Surface Dice@4 mm 0.845±0.031. Landmark localization reached median and mean errors of 7.64±0.33 px and 10.02±0.17 px, corresponding to fold-weighted millimeter errors of 2.03 mm and 2.66 mm after image-specific pixel-spacing conversion. The model produced both segmentation masks and landmark coordinates in a single forward pass, maintaining real-time inference at approximately 63 FPS. Joint segmentation of the aortic root and localization of anatomical landmarks on intraoperative fluoroscopy is feasible.

## 1 Introduction

Aortic stenosis remains one of the most common and clinically important valvular diseases in aging populations worldwide. As life expectancy increases, the number of patients with severe aortic stenosis also continues to rise. Transcatheter aortic valve implantation (TAVI) has become a major interventional treatment option in contemporary cardiology, with expanding indications in patients at intermediate and low surgical risk (1–3).

Despite this expansion, optimal valve positioning during deployment remains a major determinant of procedural success and long-term outcome. Suboptimal implantation depth and alignment relative to the annular plane and coronary ostia may increase the risk of paravalvular regurgitation, coronary obstruction, or conduction disturbances requiring permanent pacemaker implantation (4–6). Accurate real-time visualization is therefore central to safe guidance.

In current clinical practice, operators rely primarily on fluoroscopy, often supplemented by repeated contrast aortography to delineate the aortic root, annular plane, and coronary ostia. Fluoroscopy offers high temporal resolution, but it also suffers from low soft-tissue contrast, noise, respiratory and cardiac motion, and frequent occlusion by guidewires, catheters, and delivery systems. These limitations increase dependence on contrast administration and operator experience. In preoperative TAVI planning, objective CT-based quantification of the aortic annulus and related root structures has been shown to improve measurement reproducibility and support prosthesis sizing decisions (7, 8). These studies illustrate the value of software-assisted anatomical quantification, although they address preoperative CT rather than intraoperative fluoroscopic guidance. Hybrid approaches based on preoperative CT and CT-fluoroscopy fusion have been explored for procedural guidance, but their accuracy may be limited by cardiorespiratory motion, deformation from stiff guidewires, and the lack of continuous real-time updating during valve deployment (9, 10).

Recent work has begun to address the segmentation task in this setting. In a prior study by our group (11), we performed a large-scale comparison of six modern convolutional architectures for automatic aortic root segmentation on intraoperative TAVI frames. On a dataset from 80 patients with strict patient-level 5-fold cross-validation, the best single-task model, MA-Net with an EfficientNet-B4 encoder, achieved a median Dice score of 0.94 and an average symmetric surface distance (ASSD) of 4.07 mm. These results demonstrated that high-quality segmentation is feasible even under low-contrast fluoroscopic conditions. However, a segmentation mask alone does not provide the landmark coordinates needed to construct annular and sinotubular reference geometry for visual guidance.

Robot-assisted delivery platforms represent an emerging research direction in TAVI. Early translational systems include AI-guided positioning studies and the ongoing feasibility evaluation of the TAVIPILOT platform (12, 13). Integration of image-based anatomical information into such systems would require reliable landmark localization together with temporal stability, device tracking, safety constraints, and operator oversight. The present study addresses only the frame-wise image-understanding component by localizing two annular points (AA1 and AA2) and two sinotubular-junction landmarks near the coronary ostia (STJ1 and STJ2).

To address this clinical and technological need, we developed BAMNet, a multitask architecture for simultaneous aortic root segmentation and precise localization of four landmarks on intraoperative fluoroscopic images. We further hypothesized that combining global contextual reasoning, coordinate-aware feature modulation, and explicit boundary-focused supervision in a unified multitask formulation would improve both segmentation quality and landmark localization performance.

## 2 Materials and methods

### 2.1 Dataset and annotation protocol

The study used intraoperative fluoroscopic images acquired during TAVI procedures in patients with severe aortic stenosis. The present work builds on a previously published dataset of intraoperative angiographic images (14) and extends it with additional patients and revised annotations. The final dataset comprised 2,895 grayscale images of size 1,000×1,000 pixels (8-bit) obtained from 83 patients between 2018 and 2024.

The primary annotation task was binary segmentation of the aortic root. The expert mask represented the visible contour of the contrast-enhanced aortic root in the current frame. The mask includes conductors, catheters, valve delivery system components and other radiopaque instruments. Annotation was performed in the Supervisely web-based computer vision platform, consistent with the previous dataset release (Supplementary Figure S1). All segmentation masks and landmark annotations were created by a cardiovascular surgeon with experience in TAVI imaging. The annotations were subsequently reviewed by a senior cardiovascular surgeon, and ambiguous cases were discussed and corrected by consensus.

In addition to binary masks, each image was annotated with four anatomical landmarks selected to represent clinically relevant two-dimensional fluoroscopic geometry of the aortic root: two annular points (AA1 and AA2) and two sinotubular-junction landmarks near the coronary ostia (STJ1 and STJ2). AA1 and AA2 define the projected annular reference line, which serves as a two-dimensional surrogate of the virtual annular plane on fluoroscopy and is relevant for visual assessment of implantation depth. STJ1 and STJ2 provide an upper-root reference near the coronary ostial level and help define the projected aortic root axis and the relationship between the annular and sinotubular regions. For each landmark, two-dimensional coordinates (x,y) and a binary visibility flag were stored. A landmark was considered visible only when its position could be identified reliably on the current frame. When anatomy was obscured by devices, contrast opacification was insufficient, or the anatomical configuration was ambiguous, the landmark was marked as invisible. Ground-truth landmark visibility was defined by the presence of the corresponding landmark annotation. AA1 and AA2 were visible in all frames (2,895/2,895, 100.0%), STJ1 in 2,730/2,895 frames (94.3%), and STJ2 in 2,875/2,895 frames (99.3%). Overall, 11,395 of 11,580 possible landmark instances were visible (98.4%).

To assess generalization properly, all experiments followed a patient-level protocol that prevented frames from the same patient from appearing in both training and validation or test subsets. Five-fold patient-level cross-validation was used throughout. In each fold, approximately 86%–88% of patients were assigned to training and 12%–14% to validation or test. Because the number of frames per patient varied, the exact image counts differed slightly across folds.

### 2.2 Training strategy

Before model input, all images were resized to a fixed resolution of 640×640 pixels. Original single-channel fluoroscopic frames were converted to a three-channel representation by channel duplication so that an ImageNet-pretrained encoder could be used. Images were then standardized with ImageNet mean and standard deviation values. Segmentation masks were resized with nearest-neighbor interpolation to preserve binary structure. Landmark coordinates were recomputed after geometric transforms and normalized to the [0,1] range.

Augmentations were applied only to the training subset and were implemented with custom transformations. Their purpose was both to enlarge the effective training distribution and to regularize the model against overfitting. The augmentation pipeline included horizontal flipping with probability 0.5, accompanied by mandatory swapping of landmark pairs AA1↔AA2 and STJ1↔STJ2; affine transforms with scaling in the range 0.85–1.15, rotations within $\pm 20^{\circ }$, translations up to ±8% along each axis, and mild shear up to $\pm 5^{\circ }$; moderate elastic deformation; contrast-limited adaptive histogram equalization-based local contrast enhancement; random brightness and contrast adjustment; gamma correction; additive Gaussian noise; Gaussian blur and motion blur; simulated illumination non-uniformity and vignetting; and coarse dropout to mimic partial occlusion by interventional tools. Vertical flipping was intentionally excluded because it is not anatomically plausible in this fluoroscopic setting. If a landmark moved outside the image after a geometric transform, its visibility flag was reset to zero.

Training was performed in PyTorch with PyTorch Lightning. AdamW was used as the optimizer with an initial learning rate of $5\times 10^{-4}$ and weight decay of $3\times 10^{-4}$. The learning rate was adapted with ReduceLROnPlateau using a validation balance score that combined segmentation quality and landmark localization accuracy. To stabilize optimization, several parameters were scheduled smoothly. The soft-argmax temperature increased during early epochs from β=2 to β=12, enabling a transition from smoother to more localized coordinate distributions. At the same time, the width of the Gaussian target heatmaps was gradually reduced so that training started from softer targets and then shifted toward higher spatial precision.

The contribution of the landmark branch and the influence of boundary-guided feature enhancement were also introduced progressively to avoid early optimization instability. In later epochs, the weight of the point-related loss component was reduced to improve the balance between segmentation quality and landmark localization.

### 2.3 Proposed multitask architecture

BoundaryAwareMANet (BAMNet) was designed as a single multitask network for simultaneous dense segmentation of the aortic root and localization of anatomical landmarks. Both tasks are solved in one forward pass, which is important for intraoperative deployment under real-time latency constraints.

The model is built on an ImageNet-pretrained EfficientNet-V2 encoder that extracts features at multiple spatial scales. These features are fused in a decoder inspired by U-Net and MA-Net so that the network can combine global anatomical context with local contour details. At low-resolution decoder levels, additional spatial attention is applied to improve reconstruction of the continuous aortic root contour in difficult frames affected by low contrast, noise, motion, and device overlap.

The segmentation branch produces two outputs:

1. Binary segmentation logits for the aortic root;
2. An intermediate high-resolution feature representation that is reused by the landmark branch.

The landmark branch receives fused features from the deepest encoder stage and the segmentation pathway. It consists of local convolutional processing and a Coordinate Attention module that preserves vertical and horizontal positional structure. The branch predicts landmark heatmaps, while a separate auxiliary boundary head operating on segmentation features produces a boundary-weight map. Boundary guidance is implemented by projecting segmentation features and injecting them into the landmark branch during training.

**Figure 1:**
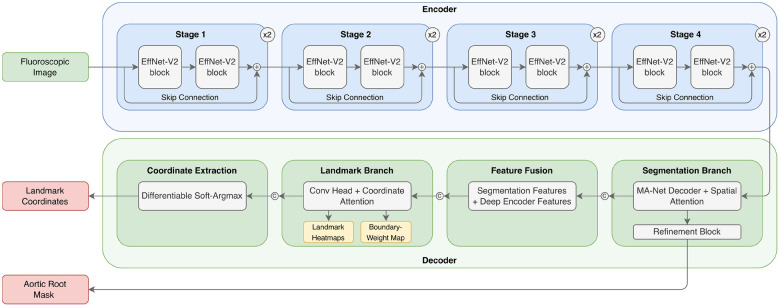
Overview of the proposed BoundaryAwareMANet (BAMNet) architecture for joint aortic root segmentation and anatomical landmark localization on fluoroscopic images. The network combines an EfficientNet-V2 encoder, a segmentation-oriented decoder, a dedicated landmark branch with Coordinate Attention, and an auxiliary boundary pathway used for boundary-guided supervision.

This design allows the model to exploit both global root morphology and local contour geometry when localizing landmarks. The complete architecture is shown in Figure 1.

### 2.4 Landmark coordinate extraction

Landmark coordinates were estimated directly from predicted heatmaps using differentiable soft-argmax, which preserved gradient flow and enabled end-to-end training. For the k-th landmark, the pixel probability at location (i,j) was defined Equation 1 as

$$P_{ij}^{\left(k\right)}=\frac{\exp\;\left(\beta H_{ij}^{\left(k\right)}\right)}{\sum_{m,n}\exp\;\left(\beta H_{mn}^{\left(k\right)}\right)},$$ (1)

where $H_{ij}^{\left(k\right)}$ is the heatmap logit and β is the temperature parameter. The landmark coordinates were then computed in Equation 2:

$${\hat{x}}_{k}=\sum_{i,j}j\;P_{ij}^{\left(k\right)},\;{\hat{y}}_{k}=\sum_{i,j}i\;P_{ij}^{\left(k\right)}.$$ (2)

This formulation provides continuous, fully differentiable coordinate estimates without a separate offset-refinement stage. In addition, segmentation features were projected into the landmark branch to softly emphasize anatomically informative regions during training.

### 2.5 Target generation

To train the landmark branch, Gaussian target heatmaps were generated from expert landmark coordinates. For landmark k, the target heatmap was defined by Equation 3 as

$$G_{k}(i,j)=\exp\;\left(-\frac{(i-y_{k}{)}^{2}+(j-x_{k}{)}^{2}}{2\sigma^{2}}\right),$$ (3)

where $\left(x_{k},y_{k}\right)$ denotes the ground-truth landmark position and σ controls the spatial width of the Gaussian target. Heatmaps were set to zero for invisible landmarks.

For the boundary branch, a separate target map was generated from the expert segmentation mask. After smoothing the mask, a Sobel-like gradient response was computed and normalized. This target explicitly emphasized the contour of the aortic root and served as an additional supervision signal.

### 2.6 Loss functions

The total loss was defined as a weighted sum of segmentation, landmark, and boundary components in Equation 4:

$$L_{total}=w_{bce}L_{bce}+w_{dice}L_{dice}+w_{\;pts}L_{\;pts}+w_{bnd}L_{bnd}.$$ (4)

In the baseline configuration, the weights were set according to Equation 5:

$$w_{bce}=1.0,\;w_{dice}=1.0,\;w_{\;pts}=0.5,\;w_{bnd}=10.0.$$ (5)

Segmentation was optimized with binary cross-entropy with logits and soft Dice loss. Landmark supervision consisted of a focal heatmap term together with a coordinate regression term defined in Equation 6:

$$L_{coord}=\frac{1}{\sum_{k}v_{k}}\sum_{k=1}^{4}v_{k}\sqrt{({\hat{x}}_{k}-x_{k}{)}^{2}+({\hat{y}}_{k}-y_{k}{)}^{2}+\varepsilon },$$ (6)

where $v_{k}$ is the visibility flag for landmark k and ε is a small constant for numerical stability. The coordinate error was computed only for visible landmarks so that the model was not penalized for frames in which anatomy could not be localized reliably.

The boundary branch was trained with Smooth L1 loss between the predicted boundary map and the target map derived from the expert segmentation mask. This term improved boundary fidelity and promoted more stable landmark localization near anatomically meaningful contours.

### 2.7 Evaluation metrics

Segmentation performance was assessed with the Dice coefficient and Intersection over Union (IoU). The Dice coefficient was defined by Equation 7 as

$$\mathrm{Dice}=\frac{2|P\cap G|}{\left|P|+|G\right|},$$ (7)

where P is the set of predicted mask pixels and G is the set of ground-truth mask pixels. Surface Dice was used as an additional contour-sensitive metric.

Landmark localization was evaluated with mean Euclidean error, median Euclidean error, and PCK@r (percentage of correct keypoints) under several error thresholds. Unless otherwise stated, r denotes a distance threshold in the 640×640 model input space and is expressed in pixels. PCK@r was defined by Equation 8 as

$$\mathrm{PCK}@r=\frac{1}{N}\sum_{k=1}^{N}I(d_{k}\leq r),$$ (8)

where $d_{k}$ is the Euclidean error for landmark k and I is the indicator function. During training, the primary point metric was mean Euclidean error over visible landmarks, whereas the final analysis also included median error, PCK, and landmark-specific performance for AA1, AA2, STJ1, and STJ2. A combined validation score that balanced segmentation quality and landmark localization error was also monitored. In the cross-validation analysis (Section 3.3), localization errors are reported in pixels at the model input resolution of 640×640 and additionally converted to millimeters using image-specific DICOM-derived pixel spacing. Because the exported annotation images were stored in the high-resolution image space, whereas BAMNet errors were measured in the 640×640 model space, the effective spacing was rescaled to the model error space before conversion. Detailed fold-wise and landmark-wise millimeter conversions are provided in Supplementary Tables S1–S2. In the baseline comparison (Section 3.1), errors are converted to millimeters using the known pixel spacing of each fluoroscopic acquisition. BAMNet always returns one coordinate for each of the four landmarks because landmark locations are obtained from four predicted heatmaps using soft-argmax. Localization failures are instead quantified using landmark error and PCK-style threshold metrics.

For statistical comparison with baseline models, paired analyses were performed at the patient level to avoid treating multiple frames from the same patient as independent observations. Frame-level metrics were first averaged within each patient, and BAMNet was then compared with each baseline using two-sided Wilcoxon signed-rank tests. Primary endpoint analyses used Dice for segmentation baselines and mean landmark error for landmark baselines; p-values were adjusted for multiple comparisons using the Holm–Bonferroni procedure. The primary paired comparisons are reported in Supplementary Table S4.

## 3 Results

### 3.1 Comparison with baseline models

BAMNet combined aortic root segmentation and landmark localization in a single inference pass. This joint output permits generation of a segmentation mask and landmark-derived geometric references without chaining separate task-specific models. Its potential use in clinician-supervised intraoperative overlays or navigation interfaces requires prospective evaluation.

Table 1 summarizes the comparison with segmentation-oriented baselines. In terms of absolute segmentation metrics, the strongest result in this table was achieved by Swin-Unet, with Dice 0.91 and IoU 0.83. Its measured throughput was approximately 2 FPS, compared with 63 FPS for BAMNet under the reported evaluation setup. BAMNet remained slightly below the best single-task segmentation baseline in these metrics, but preserved competitive segmentation quality while simultaneously solving landmark localization and providing substantially higher throughput (63 FPS). Compared with the MA-Net variant shown in the table, BAMNet demonstrated slightly higher Dice, IoU, and Surface Dice@4 mm together with better boundary agreement in terms of 95th-percentile Hausdorff distance (HD95) and ASSD. Relative to YOLO-seg-26m, BAMNet showed similar Dice and IoU, but stronger Surface Dice@4 mm and noticeably better boundary-sensitive metrics. Our previous publication (11) provides additional context for interpreting these results, as it includes a broader comparison of convolutional aortic-root segmentation architectures on intraoperative TAVI frames.

**Table 1:** Comparison of BAMNet with segmentation baselines.

Model	Dice	IoU	Surface Dice@4 mm	HD95 (mm)	ASSD (mm)	FPS
Swin-Unet	**0.91**	**0.83**	**0.89**	**8.17**	**1.95**	2
YOLO-seg-26l	0.87	0.78	0.73	55.02	8.88	25
YOLO-seg-26m	0.90	0.82	0.81	19.88	3.86	27
MA-Net	0.90	0.82	0.81	10.27	2.63	**101**
**BAMNet**	0.90	0.82	0.81	9.62	2.57	63

Evaluated on fold-1 fixed hold-out test set.

Best result is highlighted in bold.

Table 2 compares BAMNet with detection-only and keypoint-only approaches. Among single-task keypoint models, the strongest result was achieved by YOLO-keypoints-26m, with a mean localization error of 3.48 mm and a median error of 2.66 mm. BAMNet reduced the mean localization error to 3.22 mm while maintaining a comparable median error of 2.57 mm and simultaneously producing a full segmentation mask. The best median error in the table was obtained by RT-DETR (2.47 mm). BAMNet, however, achieved the lowest angle error, the lowest length error, and the highest inference speed among all evaluated methods. Within the fold-1 hold-out comparison, these results show that BAMNet provides a favorable trade-off between localization accuracy, landmark-derived geometric agreement, and computational throughput while also producing a segmentation mask.

**Table 2:** Comparison of BAMNet with landmark localization baselines.

Model	Mean err. (mm)	Median err. (mm)	Angle err. (deg)	Midpoint offset (mm)	Length err. (mm)	FPS
YOLO-detect-26l	8.32	2.71	9.45	3.29	3.31	29
YOLO-detect-26m	7.99	2.69	12.43	5.54	7.10	35
RT-DETR	7.84	**2.47**	5.84	2.39	3.92	24
YOLO-keypoints-26l	8.87	3.64	9.32	4.20	4.76	30
YOLO-keypoints-26m	3.48	2.66	5.26	**1.83**	3.03	34
**BAMNet**	**3.22**	2.57	**4.62**	2.02	**2.43**	**63**

Evaluated on fold-1 fixed hold-out test set.

Best result is highlighted in bold.

### 3.2 Qualitative visualization of BAMNet outputs

**Figure 2:**
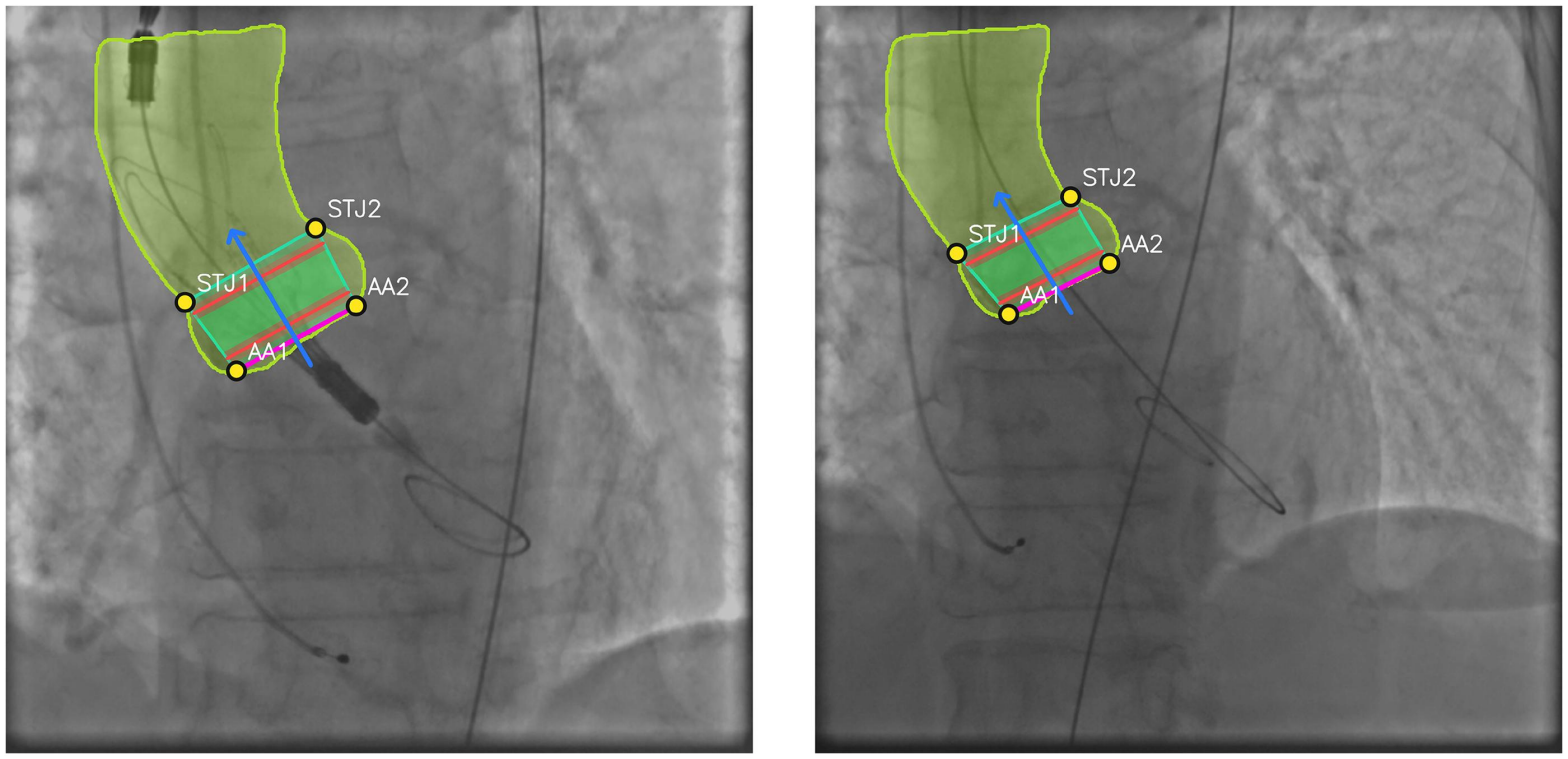
Representative BAMNet predictions on intraoperative fluoroscopic frames. The yellow contour and semi-transparent green region denote the predicted aortic root mask. Yellow points correspond to the four predicted landmarks (AA1, AA2, STJ1, STJ2). The blue arrow indicates the estimated aortic axis, and the central geometric overlay illustrates an illustrative landmark-derived implantation-zone region.

Representative BAMNet outputs on fluoroscopic frames are shown in Figure 2. In both examples, the model produces an aortic root contour together with landmarks and derived visualization primitives. The semi-transparent mask follows the annotated root region in these selected frames, while the predicted landmarks define annular and sinotubular reference lines. The display also includes a landmark-derived aortic axis and an illustrative implantation-zone overlay.

These examples demonstrate how the multitask outputs could be presented in an augmented fluoroscopic interface. They do not establish the anatomical accuracy, temporal stability, usability, or clinical benefit of the displayed overlay; these properties require dedicated quantitative and prospective evaluation.

### 3.3 Patient-level 5-fold cross-validation

To assess robustness, strict patient-level 5-fold cross-validation was performed. The corresponding fold-wise results are summarized in Table 3. Because soft-argmax always returns a coordinate for each landmark, the model produced coordinate outputs for all evaluated samples; localization quality was therefore assessed continuously using distance-based metrics rather than a discrete detection-failure count. Mean performance across folds reached a Dice score of 0.916±0.011, an IoU of 0.850±0.018, a Surface Dice@4 mm of 0.845±0.031, a median localization error of 7.64±0.33 px, and a mean localization error of 10.02±0.17 px. After image-specific conversion to millimeters in the 640×640 model error space, the overall fold-weighted median and mean landmark errors were 2.03 mm and 2.66 mm, respectively.

**Table 3:** Patient-level 5-fold cross-validation results for BAMNet.

Fold	$n_{samples}$	Dice	IoU	Surface Dice@4 mm	Median err. (px)	Mean err. (px)
1	243	0.91	0.83	0.84	8.17	10.30
2	250	0.91	0.83	0.81	7.59	9.92
3	244	0.92	0.85	0.83	7.70	9.91
4	249	0.93	0.88	0.89	7.45	10.05
5	250	0.92	0.85	0.86	7.32	9.92
Mean ± SD	247.2	0.916±0.011	0.850±0.018	0.845±0.031	7.64±0.33	10.02±0.17

The best segmentation quality was observed on fold 4, where Dice reached 0.93 and IoU 0.88. The smallest median localization error was obtained on fold 5 (7.32 px), whereas the smallest mean localization error was observed on fold 3 (9.91 px). Even the least favorable fold remained comparable to the others, indicating similar performance across the evaluated held-out patient subsets.

### 3.4 Landmark-wise localization accuracy

Landmark-specific analysis showed that AA1 was localized most accurately, with a mean error of 7.96±0.73 px and a median error of 5.92±0.40 px across folds. The most difficult point was AA2, for which the mean error reached 11.89±0.62 px and the median error 9.95±1.37 px. STJ1 and STJ2 showed intermediate performance, with STJ2 exhibiting greater inter-fold variability in mean error. After millimeter conversion, landmark-wise mean errors were 2.11 mm for AA1, 3.15 mm for AA2, 2.70 mm for STJ1, and 2.67 mm for STJ2. These values quantify frame-wise agreement with the reference annotations but do not define a threshold for clinical sufficiency. AA2 had the largest mean error and would require particular attention in any future clinician-supervised application. Detailed landmark-wise localization accuracy is reported in Supplementary Table S1.

The per-fold breakdown in Supplementary Table S2 confirms this pattern. AA2 remained the most difficult landmark in terms of absolute error, whereas STJ2 showed the greatest between-fold variability in mean error. AA1 remained the most stable in median accuracy.

### 3.5 Overall interpretation

Taken together, the results show that BAMNet provides a single-model trade-off between segmentation quality, landmark localization, and computational throughput. In the fold-1 hold-out comparison, it remained competitive with the tested segmentation baselines and achieved a lower mean landmark localization error than the tested keypoint-only and detection-only approaches. These findings concern technical performance under the reported retrospective evaluation and do not establish clinical superiority.

## 4 Discussion

This study introduced BAMNet, a multitask architecture specifically designed for simultaneous dense segmentation of the aortic root and localization of four anatomical landmarks directly on intraoperative fluoroscopic images acquired during TAVI. In patient-level 5-fold cross-validation, the model demonstrated consistent fold-wise performance, reaching Dice 0.916±0.011, IoU 0.850±0.018, Surface Dice@4 mm 0.845±0.031, median localization error 7.64±0.33 px, and mean localization error 10.02±0.17 px. These results establish the technical feasibility of joint frame-wise segmentation and landmark localization and motivate further evaluation for clinician-supervised visual guidance.

### 4.1 Technical implications and architectural contribution

The overall performance of BAMNet appears to arise not from a single isolated component but from the interaction of multiple design choices. The architecture combines an EfficientNet-V2 encoder, a modified MA-Net decoder, global spatial attention, a dedicated landmark heatmap head, an auxiliary boundary supervision pathway, and coordinate-aware feature modulation. Together, these components make it possible to maintain strong segmentation quality while also delivering clinically meaningful landmark coordinates.

The ablation study therefore focused on Position Attention in the decoder, Coordinate Attention in the landmark branch, deep-decoder feature fusion, boundary guidance and boundary loss, and the soft-argmax temperature schedule. This analysis assesses how global spatial context, coordinate-aware feature modulation, auxiliary boundary supervision, and heatmap-to-coordinate conversion affect the balance between segmentation quality and landmark localization accuracy. The contribution of each architectural component is summarized in Supplementary Figure S2. From a deployment perspective, the main practical benefit is that BAMNet removes the need to run two separate models at inference time. The reported throughput of 63 FPS on an NVIDIA RTX 4,070 demonstrates computational feasibility for real-time frame processing, although end-to-end clinical-system latency was not evaluated.

Numerical ablation results are summarized in Supplementary Table S3. The variant with a fixed soft-argmax temperature of 8 achieved the highest Dice, IoU, and Surface Dice@4 mm, indicating particularly strong segmentation performance. However, the full BAMNet configuration yielded the lowest mean and median landmark localization errors together with the best PCK@10, and therefore provided the most balanced combination of segmentation quality and landmark localization accuracy. All ablation experiments were conducted on the fold-1 hold-out test set; cross-validated ablation was not performed due to computational cost.

### 4.2 Clinical relevance

BAMNet generates an aortic root segmentation mask and four anatomical landmark coordinates within a single forward pass. In patient-level five-fold cross-validation, the fold-weighted median and mean landmark localization errors were 2.03 mm and 2.66 mm, respectively. The landmark-wise mean errors ranged from 2.11 mm for AA1 to 3.15 mm for AA2. On the fold-1 fixed hold-out test set used for baseline comparison, the corresponding median and mean errors were 2.57 mm and 3.22 mm. Together, these results demonstrate reproducible frame-wise localization and support further evaluation of BAMNet as a clinician-supervised anatomical overlay and pre-positioning aid. They do not, however, establish the precision or temporal stability required for autonomous valve deployment.

The predicted annular and sinotubular-junction landmarks provide a compact two-dimensional representation of aortic root geometry that may assist operators in assessing valve position relative to the visible anatomy. Implantation depth and alignment are clinically important because they are associated with paravalvular regurgitation, coronary obstruction, and conduction disturbances requiring permanent pacing (4–6). Nevertheless, BAMNet was evaluated retrospectively on individual frames and was not used to guide valve deployment. Its effect on positioning accuracy, procedural decisions, or clinical outcomes therefore remains to be established prospectively.

A further potential application is to support visualization during procedural phases that currently require repeated contrast aortography. Reducing the number or volume of contrast injections could be clinically relevant because renal complications remain an important concern in the TAVI population (15, 16). However, BAMNet was trained and evaluated on contrast-enhanced fluoroscopic images, and the present study did not measure contrast volume or determine whether the model can maintain useful visualization between contrast injections. Any reduction in contrast use should therefore be considered a testable hypothesis rather than a demonstrated benefit.

A reduction in additional angiographic acquisitions could also decrease radiation exposure to patients and operating-room personnel. Radiation dose, fluoroscopy time, and the number of angiographic runs were not recorded in this study. Consequently, the relationship between BAMNet-assisted visualization and radiation burden must be evaluated in a prospective workflow study rather than inferred from the current frame-wise results.

CT-based planning provides patient-specific three-dimensional information for annular assessment, C-arm angle selection, and evaluation of coronary obstruction risk. Objective and automatic CT-based quantification of aortic annulus geometry has previously been shown to improve measurement reproducibility and reduce interobserver variability in TAVI planning (7, 8). CT–fluoroscopy fusion can further register this preoperative information for intraoperative guidance, but its accuracy may be affected by registration error, cardiorespiratory motion, and deformation induced by guidewires or delivery systems (9, 10). BAMNet addresses a complementary task by processing the currently visible two-dimensional fluoroscopic anatomy without requiring CT-to-fluoroscopy registration at runtime. Its reported throughput of 63 FPS demonstrates computational feasibility for real-time frame processing, but not yet real-time clinical guidance.

BAMNet should therefore be considered a potential complement to preprocedural CT planning and fusion-based guidance rather than a replacement for these methods. Prospective evaluation should determine whether clinician-supervised use improves positioning accuracy or reduces contrast volume, radiation exposure, procedure duration, or complication rates. Temporal filtering, motion compensation, device tracking, and continuous operator verification would also be required before integration into robot-assisted or closed-loop navigation systems.

### 4.3 Toward visual assistance and robot-assisted TAVI guidance

One potential application of BAMNet is clinician-supervised visual assistance during TAVI. A segmentation mask delineates the visible root region, whereas landmark coordinates permit construction of annular and sinotubular reference lines and a projected root axis. The multitask output therefore provides more explicit two-dimensional geometric information than segmentation alone. Whether this information improves operator interpretation or positioning decisions remains to be tested prospectively.

The present study does not evaluate robotic control, closed-loop navigation, or autonomous valve deployment. Any future use in robot-assisted systems would require additional components, including temporal filtering, device tracking, motion compensation, safety constraints, prospective validation, and continuous operator oversight.

In patient-level 5-fold cross-validation, the median landmark localization error was 7.64±0.33 px and the mean error was 10.02±0.17 px, providing an estimate across the evaluated held-out patient subsets. Temporal modeling, sequence-level filtering, and explicit compensation for cardiac and respiratory motion may reduce frame-to-frame coordinate variation, but this hypothesis was not tested. BAMNet was designed to combine local appearance with global spatial context; however, performance under specific degrees of device overlap or anatomical occlusion was not evaluated separately.

### 4.4 Limitations

This study has several limitations. First, despite the relatively large dataset of 2,895 images from 83 patients, the material remains single-center. External validation on data from other centers, fluoroscopy systems, and valve types is needed before broader generalization can be claimed. Second, all analysis was performed in a frame-wise setting; temporal consistency across neighboring frames was not studied. That limitation is particularly important for future integration into robotic systems.

Third, a further limitation is the absence of a direct benchmark against general-purpose frameworks such as nnU-Net for segmentation and nnLandmark-like networks for landmark localization. nnU-Net provides a strong self-configuring baseline for medical image segmentation, whereas landmark-specific networks provide relevant reference methods for coordinate prediction. The current BAMNet implementation combines aortic-root segmentation, landmark localization, training-time masking of invisible landmarks, and high-throughput fluoroscopic inference in a single forward pass. Future studies should include a direct comparison with these methods under the same patient-level cross-validation protocol.

Fourth, BAMNet does not estimate landmark visibility or prediction reliability at inference time. Soft-argmax always returns a coordinate, including in frames in which a landmark may be obscured or poorly localized; confidence estimation or an explicit non-detection mechanism should therefore be evaluated. Fifth, the observed localization errors cannot be interpreted as sufficient for autonomous control, which would additionally require predictable temporal behavior and system-level safety validation. Finally, we have not performed a prospective clinical evaluation of the effect of BAMNet on positioning accuracy, procedure duration, contrast volume, radiation dose, or complication rates.

### 4.5 Future directions

Several extensions follow from the present work. The first is a move toward multi-frame modeling with temporal loss functions or recurrent or video architectures in order to stabilize landmark tracking throughout the cardiac cycle. A second direction is biplane fluoroscopy with 3D reconstruction of anatomical landmarks. A third is prospective clinical evaluation with explicit measurement of positioning accuracy, procedure duration, contrast use, and radiation exposure. Finally, an important translational direction is integration of BAMNet into a clinician-supervised human–machine guidance loop. Any subsequent evaluation in robot-assisted TAVI would require continuous operator oversight and independent system-level safety validation.

## 5 Conclusion

BoundaryAwareMANet (BAMNet) is a multitask deep learning architecture for simultaneous aortic root segmentation and localization of four key anatomical landmarks on intraoperative fluoroscopic images acquired during TAVI. Unlike single-task formulations, BAMNet produces both a dense anatomical mask and a set of geometrically interpretable landmarks within a single forward pass. This unified output provides two-dimensional reference structures for the aortic annulus and sinotubular junction without requiring sequential application of separate segmentation and landmark-localization models.

In patient-level 5-fold cross-validation, BAMNet demonstrated consistent fold-wise performance, achieving Dice 0.916±0.011, IoU 0.850±0.018, and Surface Dice@4 mm 0.845±0.031. The median and mean landmark localization errors were 7.64±0.33 px and 10.02±0.17 px, respectively. After image-specific conversion using DICOM-derived pixel spacing, the fold-weighted median and mean errors were 2.03 mm and 2.66 mm, respectively. These results establish the technical feasibility of joint frame-wise segmentation and landmark localization across different patient subsets.

BAMNet may therefore provide a technical foundation for the further development of clinician-supervised anatomical overlays and visual guidance systems for TAVI. However, this retrospective study does not demonstrate clinical benefit, temporal stability, reduced reliance on repeated contrast aortography, or suitability for semi-autonomous or autonomous valve deployment. Further work should include external multicenter validation, evaluation of temporal consistency across image sequences, estimation of prediction reliability, and prospective assessment of positioning accuracy, contrast use, radiation exposure, and workflow effects. Integration into robot-assisted or closed-loop navigation systems should be considered only with continuous clinician oversight and independent system-level safety validation.

## Data Availability

The datasets presented in this study can be found in online repositories. The names of the repositories and their corresponding access information are provided below: • Source code: https://github.com/Nikita75699/BAMNet • Dataset: https://doi.org/10.5281/zenodo.19219901 • Models: https://doi.org/10.5281/zenodo.19295876
