# Supplementary material for: Joint aortic root segmentation and landmark localization on intraoperative fluoroscopy for TAVI guidance
Source: Front Cardiovasc Med. 2026 Jul 15;13:1886469. doi: 10.3389/fcvm.2026.1886469 (PMC13416090; doi:10.3389/fcvm.2026.1886469)
Supplement: Supplementary file 1 [file Datasheet1.pdf]

Supplementary Material

1 SUPPLEMENTARY FIGURES

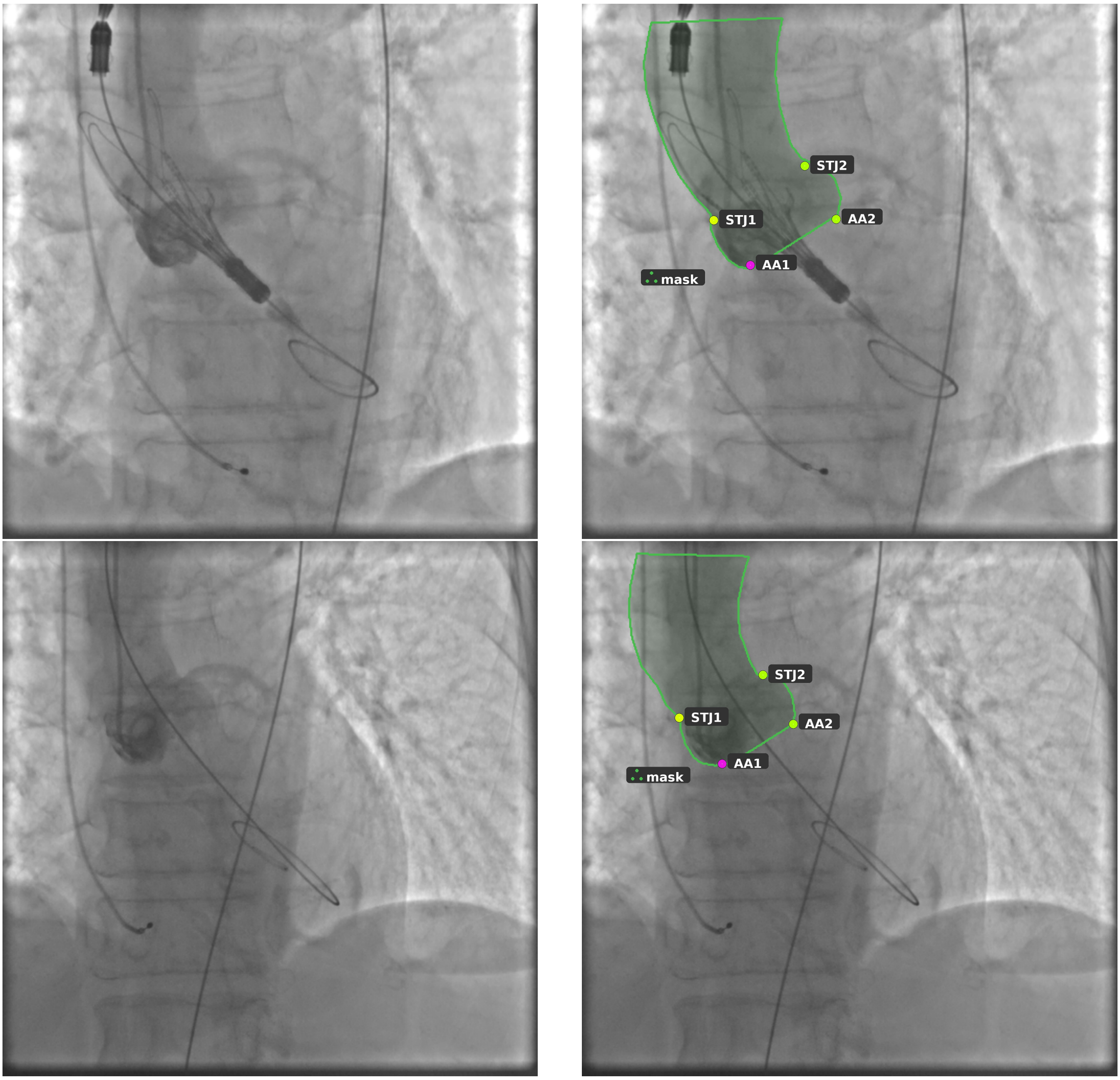

Figure S1. Representative fluoroscopic images (a, c) and corresponding expert annotations (b, d) illustrating the aortic root mask and four anatomical landmarks (AA1, AA2, STJ1, STJ2).

2 SUPPLEMENTARY TABLES

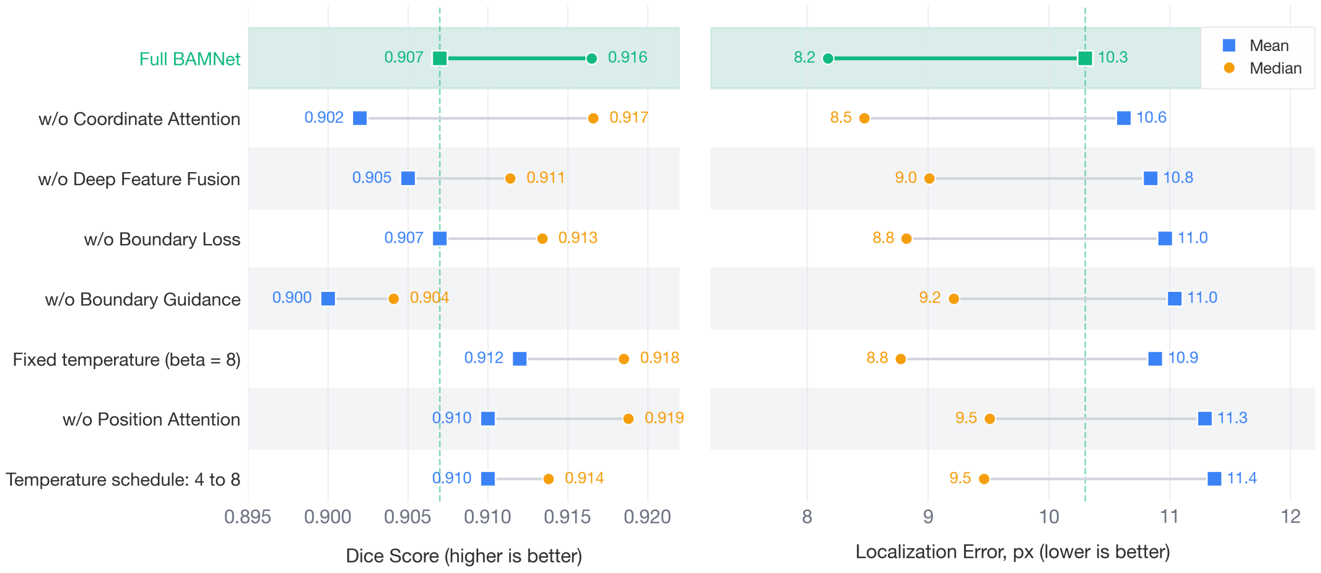

Figure S2. Summary of the BAMNet ablation study across the main architectural variants. The figure highlights how global spatial attention, coordinate-aware modulation, feature fusion, boundary-related supervision, and the soft-argmax configuration affect the balance between segmentation quality and landmark localization accuracy.

Table S1. Landmark-wise localization accuracy aggregated over five folds. Pixel errors are reported in the 640 $\times$ 640 model error space; millimeter errors were computed using image-specific DICOM-derived spacing rescaled to the same space.

Landmark	Mean err. (px)	Median err. (px)	Std. err. (px)	Mean err. (mm)	Median err. (mm)
AA1	7.96 ± 0.73	5.92 ± 0.40	7.66 ± 1.61	2.11 ± 0.16	1.57 ± 0.09
AA2	11.89 ± 0.62	9.95 ± 1.37	8.60 ± 1.00	3.15 ± 0.16	2.64 ± 0.36
STJ1	10.20 ± 0.87	7.66 ± 0.14	8.86 ± 2.20	2.70 ± 0.18	2.03 ± 0.06
STJ2	10.02 ± 1.46	7.73 ± 0.93	8.28 ± 2.01	2.67 ± 0.46	2.06 ± 0.31

Table S2. Per-fold landmark localization errors for BAMNet after pixel-to-millimeter conversion. Values are reported as mean / median error in millimeters for each landmark.

Fold	AA1 mean / median (mm)	AA2 mean / median (mm)	STJ1 mean / median (mm)	STJ2 mean / median (mm)
1	2.13 / 1.53	3.39 / 3.19	2.50 / 1.96	2.79 / 2.01
2	1.90 / 1.50	3.19 / 2.56	2.56 / 2.11	3.42 / 2.60
3	2.33 / 1.64	3.16 / 2.74	2.71 / 2.04	2.33 / 1.91
4	2.16 / 1.68	3.11 / 2.48	2.94 / 2.04	2.33 / 1.82
5	2.02 / 1.48	2.93 / 2.22	2.80 / 2.01	2.46 / 1.94

Table S3. Numerical results of the BAMNet ablation study across the main architectural variants. Evaluated on fold-1 fixed hold-out test set.

Variant	Dice	IoU	Surface Dice@4 mm	Mean err. (px)	Median err. (px)	PCK@10 (%)	FPS
Full	0.91	0.83	0.84	**10.30**	**8.17**	**59.88**	63
No Pos Attn	0.91	0.84	0.83	11.29	9.51	52.98	**69**
No Coord Attn	0.90	0.83	0.83	10.62	8.47	57.41	67
No Fusion	0.91	0.83	0.83	10.84	9.01	55.76	65
No Bnd Guid	0.90	0.82	0.82	11.04	9.21	53.70	66
No Bnd Loss	0.91	0.84	0.83	10.96	8.82	54.73	64
Fixed 8	**0.91**	**0.84**	**0.84**	10.88	8.77	56.69	66
Sched 4→8	0.91	0.84	0.83	11.37	9.46	53.29	66

Table S4. Patient-level paired Wilcoxon signed-rank tests for the primary endpoints with Holm–Bonferroni correction. The segmentation endpoint was Dice, whereas the landmark endpoint was mean landmark error in millimeters. Metrics were first averaged within each patient.

Endpoint	Model 1	Model 2	Raw p	Holm p	Significant ($\alpha = 0.05$)
Segmentation	BAMNet	MA-Net	7.81×10^{-2}	1.56×10^{-1}	No
Segmentation	BAMNet	Swin-Unet	7.81×10^{-3}	3.13×10^{-2}	Yes
Segmentation	BAMNet	YOLO-seg-26l	7.81×10^{-3}	3.13×10^{-2}	Yes
Segmentation	BAMNet	YOLO-seg-26m	3.13×10^{-1}	3.13×10^{-1}	No
Landmark	BAMNet	RT-DETR	2.34×10^{-2}	4.69×10^{-2}	Yes
Landmark	BAMNet	YOLO-detect-26l	7.81×10^{-3}	3.91×10^{-2}	Yes
Landmark	BAMNet	YOLO-detect-26m	7.81×10^{-3}	3.91×10^{-2}	Yes
Landmark	BAMNet	YOLO-keypoints-26l	7.81×10^{-3}	3.91×10^{-2}	Yes
Landmark	BAMNet	YOLO-keypoints-26m	2.34×10^{-2}	4.69×10^{-2}	Yes
